# *Aspergillus* Endocarditis in Native Valves in Non-Traditional Hosts: A Systematic Review of a Case in a Patient with CREST Syndrome and Advanced Liver Cirrhosis

**DOI:** 10.3390/jof11120836

**Published:** 2025-11-26

**Authors:** Leticia Espinosa-del-Barrio, Elia Gómez G. de la Pedrosa, Noelia Álvarez-Díaz, Javier Guzmán Martínez, María Dolores Corbacho Loarte, Rosa Escudero Sánchez, Pilar Martín-Dávila, Jesús Fortún Abete, Javier Cobo Reinoso, Vicente Pintado García, Francesca Gioia

**Affiliations:** 1 Internal Medicine Department, Sant Joan d’Alacant University Hospital, 03550 Sant Joan d’Alacant, Spain; guzmanjavier1994@gmail.com; 2 Clinical Microbiology Department, Ramón y Cajal University Hospital, 28034 Madrid, Spain; elia.gomez@gmail.com; 3 Medical Library, Ramón y Cajal University Hospital, 28034 Madrid, Spain; nalvarezd@salud.madrid.org; 4 Infectious Diseases Department, Ramón y Cajal University Hospital, 28034 Madrid, Spain; mdcorbacho@hotmail.com (M.D.C.L.); rosa.escudero@salud.madrid.org (R.E.S.); pmartindav@gmail.com (P.M.-D.); fortunabete@gmail.com (J.F.A.); javier.cobo@salud.madrid.org (J.C.R.); vicente.pintado@salud.madrid.org (V.P.G.); francesca_gioia@hotmail.com (F.G.)

**Keywords:** *Aspergillus fumigatus*, native valve endocarditis, fungal endocarditis, systematic review, immunocompetent host, tissue-based diagnosis

## Abstract

**Background**: *Aspergillus* endocarditis is a rare but life-threatening form of infective endocarditis that typically occurs in patients with a history of cardiac surgery, prosthetic valve implantation, or profound immunosuppression. Native valve involvement in non-traditional hosts remains exceptionally rare and is diagnostically challenging. **Case presentation**: We describe a 56-year-old woman with CREST syndrome and advanced liver disease awaiting transplantation who developed native aortic valve endocarditis. Blood cultures and serum biomarkers (galactomannan and β-d-glucan) were also negative. Transthoracic echocardiography revealed vegetation on the aortic valve. Valve replacement was performed, and *Aspergillus fumigatus* was isolated from two valve cultures. Liposomal amphotericin B was initiated; however, the patient died of multiorgan failure two weeks later. **Systematic review**: To contextualise this case, we conducted a systematic review of the literature following the PRISMA guidelines. We included microbiologically confirmed cases of native valve *Aspergillus* endocarditis based on valve or embolic tissue analysis. Forty-three studies met the inclusion criteria, comprising 45 patients in total. Data were independently extracted by two reviewers and narratively synthesised due to clinical heterogeneity. **Conclusions**: This case illustrates the diagnostic and therapeutic challenges of native-valve *Aspergillus* endocarditis in patients without classical risk factors for the disease. Early imaging and a high index of suspicion are crucial for diagnosis. Combined surgical and antifungal therapy remains the cornerstone of management, although the mortality rate remains high.

## 1. Introduction

Fungal endocarditis accounts for approximately 1–6% of all cases of infective endocarditis and remains a life-threatening condition, with mortality rates exceeding 50% even under optimal therapy [1]. *Candida* species are the most frequently implicated pathogens, followed by *Aspergillus* spp., which are responsible for up to one-quarter of fungal endocarditis cases [1].

*Aspergillus* endocarditis (AE) typically occurs in patients with a history of cardiothoracic surgery, prosthetic heart valves, cardiac implantable devices, intravenous drug use, or profound immunosuppression [1,2,3]. In recent years, novel risk factors such as influenza, COVID-19, and chimeric antigen receptor T-cell (CAR-T) therapy have expanded the at-risk population, suggesting that AE may also affect patients with transient or non-classical immune dysfunction [4,5,6].

Diagnosis is often delayed due to nonspecific clinical presentation and low microbiological yield. Blood cultures are usually negative, and serum biomarkers such as galactomannan (GM) and (1,3)-β-d-glucan (BDG) have limited sensitivity in non-neutropenic hosts, particularly after antifungal exposure [1,7,8]. Although echocardiography is essential for detecting vegetations, microbiological confirmation is frequently achieved only through culture or histopathological analysis of excised valves or embolic material [1,3]. Current international guidelines recommend early surgical intervention combined with systemic antifungal therapy as the cornerstone of management [9,10].

We describe a rare case of native aortic valve endocarditis caused by *Aspergillus fumigatus* in a patient with CREST syndrome and advanced liver cirrhosis, two comorbidities not previously recognised as predisposing factors for AE. Both conditions are associated with endothelial dysfunction and immune dysregulation, which may facilitate the fungal invasion. This unusual presentation, occurring outside classical immunosuppression settings, prompted us to conduct a systematic review to better characterise the contemporary landscape of native valve *Aspergillus* endocarditis.

## 2. Case Presentation

A 56-year-old woman with a history of primary biliary cirrhosis complicated by a transjugular intrahepatic portosystemic shunt (TIPS) placed one year earlier was on the waiting list for liver transplantation. The patient also had CREST syndrome with prominent cutaneous and vascular involvement, which was treated with hydroxychloroquine.

She was admitted with decompensated cirrhosis, persistent fever, anasarca, and progressive functional decline. Initial laboratory tests revealed neutrophil-predominant leukocytosis and elevated inflammatory markers. The repeated blood cultures remained sterile despite persistent fever. Urine cultures yielded extended-spectrum beta-lactamase (ESBL)-producing *Escherichia coli* and *Klebsiella pneumoniae* and empirical antibiotic therapy was initiated with piperacillin–tazobactam and fosfomycin.

**Figure 1:**
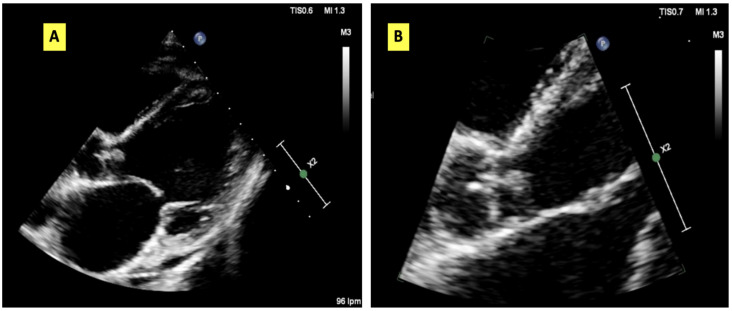
Aortic vegetation visualised using transthoracic echocardiography. (**A**) 3-chamber apical plane. (**B**) Enlarged image of the vegetation.

Owing to persistent fever and systemic inflammation despite therapy, transthoracic echocardiography (TTE) was performed, revealing a mobile vegetation measuring 1.0 × 0.5 cm on the right coronary cusp of the aortic valve, with associated severe aortic regurgitation (Figure 1). Antimicrobial therapy was escalated to meropenem (1 g every 8 h) and daptomycin (10 mg/kg/day) therapy. Cardiothoracic surgery was urgently performed.

The patient underwent aortic valve replacement with a biological prosthesis. Intraoperative specimens revealed *Aspergillus fumigatus* in two separate valve cultures. Serum galactomannan and (1,3)-β-d-glucan were both negative. PCR assays targeting panfungal, panbacterial, and *Aspergillus* DNA in the blood were also negative.

Given the patient’s advanced liver disease, antifungal therapy with liposomal amphotericin B (5 mg/kg/day) was initiated on the day that the Aspergillus culture test results came back positive. Despite receiving appropriate antifungal treatment and intensive supportive care, her condition deteriorated, and she died of multiple organ failure 14 days after surgery and 17 days after we started the antifungal treatment.

## 3. Material and Methods

We conducted a systematic review of published cases of native valve endocarditis (NVE) caused by *Aspergillus* spp. following the 2020 PRISMA guidelines. The protocol was designed to ensure diagnostic robustness by including only microbiologically confirmed cases based on the tissue analysis.

A comprehensive literature search was performed in PubMed/MEDLINE and Embase, covering all records from inception to 4 March 2025. The search strategy combined MeSH/Emtree terms and free-text keywords for “*Aspergillus fumigatus*” and “endocarditis.” No language or publication date restrictions were imposed. The full search strategy is presented in Appendix A. All references were imported into Rayyan, where the duplicates were automatically identified and removed. Two independent reviewers (LE and FG) screened all the titles and abstracts. The selected full texts were assessed for eligibility. Discrepancies were resolved by consensus, and a third reviewer (VP) was consulted when required. Zotero was used for full-text organization, reference management, and citation tracking.

We included case reports and case series describing native valve endocarditis caused by *Aspergillus* spp., confirmed by culture, histopathology, or PCR of valve tissue, vegetations, or embolic material. Conference abstracts and posters were included if sufficient clinical and microbiological data were available for them.

We excluded cases with prosthetic valves, intracardiac devices, transplanted hearts, or polymicrobial infections in which *Aspergillus* was not the primary pathogen. We also excluded fungal aortitis, mural endocarditis, myocardial aspergillosis without valvular involvement, and cases based solely on blood polymerase chain reaction (PCR) or serum biomarkers (e.g., galactomannan or BDG). Aggregated case series without individual-level data were excluded from the study.

Data extraction was performed independently and in duplicate using predefined templates. The extracted variables included demographics, underlying conditions, immune status, clinical presentation, diagnostic imaging, microbiological findings, antifungal treatment, surgical intervention, and outcomes. Discrepancies were resolved through discussion. Given the rarity and heterogeneity of the condition, a narrative synthesis of the findings was conducted.

## 4. Results

**Figure 2:**
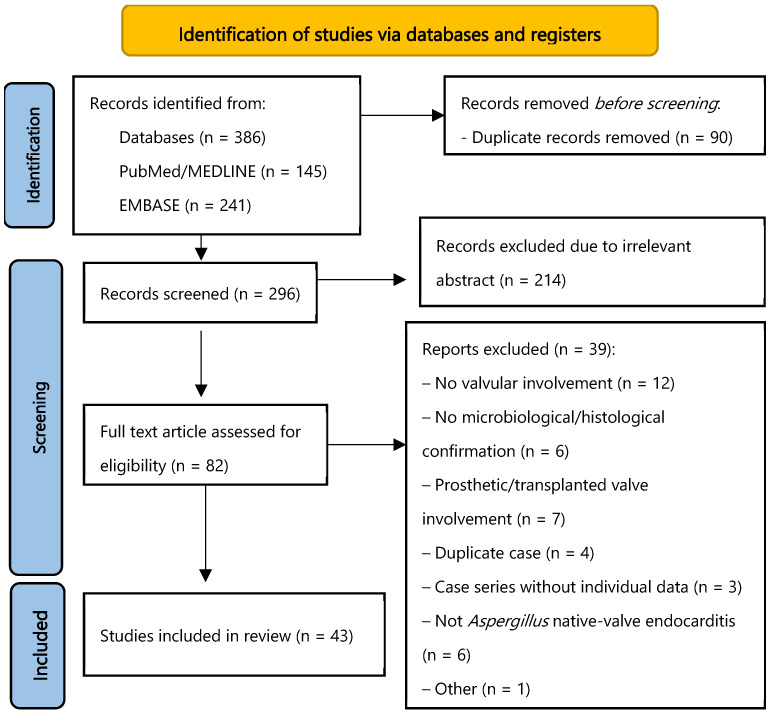
Study Selection. PRISMA flow diagram.

The study selection process is summarised in Figure 2. Of the 43 included studies, 40 were individual case reports, and three were case series that described multiple patients. Thirty-seven studies (86%) were published as full-text articles in peer-reviewed journals, and six (14%) were available only as conference abstracts. The included studies originated from 18 countries across four continents, with the highest representation from Europe (n = 17), followed by Asia (n = 12), North America (n = 10), and Oceania (n = 2). The publication timeline spans nearly six decades, from 1968 to 2025. An apparent increase in reported cases has been noted in recent years, with 12 studies published in the 2010s and 14 in the 2020s (through March 2025). The earlier decades contributed fewer reports, including 10 in the 2000s, six in the 1990s, and one in the 1960s. A detailed summary of the included studies is presented in Table 1.

**Figure 3:**
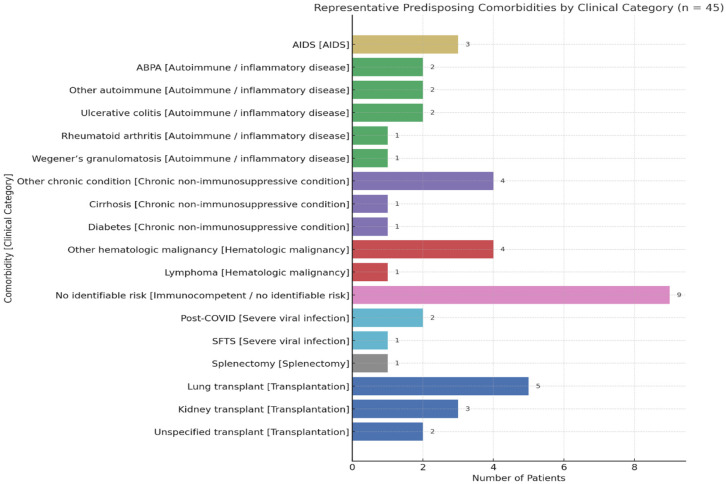
Distribution of representative predisposing comorbidities in 45 patients with *Aspergillus* native valve endocarditis. Each patient was classified into a single clinical category based on the primary predisposing condition. Categories are color-coded as follows: Blue: solid organ transplantation (kidney, lung, unspecified), Green: autoimmune or inflammatory disease (e.g., ulcerative colitis, Wegener’s, ABPA, rheumatoid arthritis), Red: haematologic malignancy, Purple: chronic non-immunosuppressive condition (e.g., cirrhosis, diabetes), Yellow: HIV-related immunosuppression, Cyan: severe viral infection (post-COVID-19, SFTS), Grey: splenectomy, Pink: no identifiable risk (immunocompetent).

Forty-five patients met the inclusion criteria and were included in the final analysis. The median age was 53 years (IQR, 35–60 years), ranging from 17 to 79 years. A male predominance was observed (n = 30, 66.7%). The most common predisposing conditions were solid organ transplantation (n = 10, 20.8%) and autoimmune or inflammatory diseases (n = 10, 20.8%). Haematologic malignancies and chronic non-immunosuppressive conditions, such as cirrhosis, diabetes, or COPD, were identified (n = 6, 12.5% each). AIDS-related immunosuppression and severe viral infections accounted (n = 3, 6.2% each), and a patient (n = 1, 2.1%) had a history of post-traumatic splenectomy. Nine patients (18.8%) were immunocompetent without identifiable risk factors. The distribution of specific underlying conditions within each clinical category is shown in Figure 3.

To further characterise the host status, we analysed the immunosuppressive regimens administered to the included patients. Corticosteroids were the most commonly used agents (n = 17, 37.8%), often in combination with other immunosuppressive therapies. Two patients received corticosteroids alongside multiple immunosuppressive agents, and three were treated with a combination of corticosteroids and chemotherapy. Chemotherapy alone was administered to two patients (4.4%). Eleven patients (24.4%) received multi-agent immunosuppression, including calcineurin inhibitors, antimetabolites, and antithymocyte globulin. Notably, in 10 patients (22.2%), no pharmacological immunosuppression was identified, and in six cases (13.3%), the specific drugs, if any, were not reported.

The mitral valve was the most frequently affected site (n = 27, 60.0%), followed by the aortic valve (n = 13, 28.9%) and the tricuspid valve (n = 5, 11.1%).

Transthoracic echocardiography (TTE) alone was the most frequently used modality, documented in 12 patients (26.7%), followed by a combined approach using TTE and transesophageal echocardiography (TEE) in 11 patients (24.4%). Multimodal imaging, including TTE, TEE, computed tomography (CT), and magnetic resonance imaging (MRI), was performed in six patients (13.3%). Five patients (11.1%) were evaluated exclusively using advanced cross-sectional imaging (CT/MRI), and in five additional 5 cases (11.1%), the diagnosis was made intraoperatively without prior imaging. TEE alone was used in two patients (4.4%), and postmortem diagnosis was reported in three cases (6.7%). The imaging modality was not explicitly specified in one patient (2.2%).

Microbiological confirmation was obtained using several diagnostic methods. The most common method was combined histopathology and fungal culture from the valve or embolic tissue (n = 26, 57.8%). Culture alone was used in 11 cases (24.4%), and culture plus PCR was used in three cases (6.7%). Histopathology without culture was reported in two patients (4.4%), while one case (2.2%) was confirmed by PCR alone of valve tissue. One additional case was diagnosed using histopathology and PCR, and another using a combination of histology, culture, and PCR, all from valvular or embolic samples.

Among the 45 patients, *Aspergillus* species were identified in 40 cases (88.9%). *A. fumigatus* was the predominant species (n = 38, 84.4%), followed by *A. flavus* (n = 1, 2.2%), and *A. terreus* (n = 1, 2.2%). In five cases (11.1%), the pathogen was identified only at the genus level (*Aspergillus*). No infections caused by the cryptic species were documented.

Valve surgery was performed in the majority of cases. Valve replacement was performed in 28 patients (62.2%), and valve repair without prosthetic implantation was performed in three (6.7%). Thirteen patients (28.9%) did not undergo surgery, and in one case (2.2%), the surgical status was not documented. Four patients (8.9%) required surgical embolectomy, which was performed concurrently with mitral valve replacement.

Antifungal therapy was administered in 42 of the 45 patients (93.3%). Azole monotherapy, predominantly with voriconazole, was used in nine patients (20.0%), whereas eight (17.8%) received amphotericin B alone. Combination regimens included azoles plus amphotericin B (n = 12, 26.7%), azoles plus echinocandins (n = 4, 8.9%), and amphotericin B plus an echinocandin (n = 3, 6.7%). Sequential therapy was reported in two cases: amphotericin B followed by itraconazole (n = 1) and fluconazole followed by amphotericin (n = 1). Three patients (6.7%) received no antifungal therapy and were diagnosed postmortem. Treatment details were not reported in four cases (8.9%).

Overall, 28 patients (62.2%) died during hospitalisation or follow-up, whereas 15 (33.3%) survived, including several patients with residual morbidity.

## 5. Discussion

This case illustrates a rare but increasingly recognised phenotype of native-valve *Aspergillus* endocarditis (NVAE) in a non-neutropenic, non-transplanted patient without prior cardiac surgery or overt pharmacological immunosuppression. Although this clinical profile falls outside the traditional risk categories outlined in the current guidelines [1,9], it aligns with an emerging body of evidence documenting NVAE in patients with complex inflammatory or metabolic comorbidities [1]. The coexistence of CREST syndrome and advanced liver cirrhosis in this case provides a biologically plausible substrate for invasive fungal disease, given the documented roles of both conditions in promoting endothelial dysfunction and impairing innate immunity [53].

Our systematic review supports a broader understanding of host susceptibility. Several cases have been reported in patients with chronic, traditionally non-immunosuppressive diseases, such as cirrhosis, diabetes, and COPD, which may generate low-grade but persistent immune dysregulation [1]. In addition, post-viral immune remodelling has emerged as a relevant risk factor, particularly following severe respiratory infections, such as COVID-19 [4,5,54]. These findings challenge the binary framework of immunocompetent versus immunocompromised hosts and suggest that immune vulnerability may be more dynamic and context-dependent than previously appreciated.

Notably, recent case reports, such as that of Hosseini et al. [5], describe probable NVAE following COVID-19 in patients without classical risk factors based on clinical and blood-based microbiological findings. However, such cases were not included in our review because of the absence of microbiological confirmation from valvular or embolic tissue, as required by our predefined inclusion criteria.

This case exemplifies the diagnostic challenges associated with NVAE. Molecular identification was not performed, which indeed limits confirmation at the species level. However, two independent valve cultures yielded colonies with the characteristic macroscopic and microscopic morphology of Aspergillus fumigatus, providing strong diagnostic evidence. According to current international guidelines (IDSA 2016; ECMM/ISHAM 2021) and recent comprehensive reviews [1], culture or histopathological confirmation from valvular tissue remains the diagnostic gold standard for fungal endocarditis, whereas molecular techniques, although valuable, are not universally available and show variable sensitivity depending on tissue quality and fungal burden.

Negative blood cultures and the limited performance of fungal biomarkers are frequent in non-neutropenic patients [10,53]. As in many reported cases, echocardiography, often prompted by persistent fever or embolic phenomena, was crucial for detecting valvular vegetations, while definitive diagnosis required surgical analysis of the tissue. These observations reinforce the central role of imaging and tissue-based microbiology in establishing a diagnosis, particularly in the absence of reliable serological markers [7,10].

Treatment in this patient was further complicated by hepatic dysfunction, which precluded the use of first-line azoles and required the initiation of liposomal amphotericin B. Despite timely surgery and antifungal therapy in accordance with current recommendations, the patient died shortly after the intervention, a pattern observed in many similar cases [53]. The fatal outcome precluded evaluation of long-term management and prognostic factors. However, this finding aligns with published series [1,8] reporting mortality rates exceeding 50–70% despite combined surgical and antifungal therapy. This case illustrates the fulminant course and poor prognosis of *Aspergillus* endocarditis in patients with advanced liver disease, emphasizing the extremely narrow therapeutic window and the need for early recognition and coordinated multidisciplinary management. This highlights the limited therapeutic window and underscores the need for tailored management strategies in patients with multisystem diseases.

The included studies span from 1968 to 2025, which may introduce temporal heterogeneity owing to major advances in imaging, surgical techniques, and antifungal therapy. Nevertheless, the majority of cases were published after 2010, reflecting modern diagnostic and therapeutic practices. While these differences may influence individual outcomes, they also highlight the progressive improvement in the recognition and management of native-valve *Aspergillus* endocarditis.

By synthesising data from 45 microbiologically confirmed cases, this review provides a structured overview of the clinical spectrum of NVAE. It identifies consistent challenges across diverse settings: delayed diagnosis, frequent reliance on intraoperative specimens, heterogeneous antifungal regimens and suboptimal use of fungal diagnostics. These findings support recent calls for more standardised diagnostic approaches and emphasise the value of advanced molecular tools in analysing tissue specimens [55].

The quality and completeness of the published reports varied, with occasional omissions regarding immune status, imaging modality, antifungal regimen, or follow-up. Because of the extreme rarity of native-valve *Aspergillus* endocarditis, conference abstracts were also included when they provided sufficient diagnostic and clinical information, as this strategy reduced publication bias while maintaining diagnostic rigor. Nevertheless, incomplete reporting in some studies remains an inherent limitation of this review.

A key strength of this study is the integration of a rigorously documented clinical case with a systematic review restricted to culture-, histology-, or PCR-confirmed NVAE cases. This dual approach offers both granularity and generalisability to the model. However, our study had some limitations. The synthesis is subject to publication bias, lacks longitudinal outcome data, and is limited by incomplete reporting of the immunological status and diagnostic procedures. Advanced methods, such as valve PCR or metagenomics, remain underutilised [55]. The predominance of tissue-proven and surgically treated cases likely reflects a bias in publications towards histologically confirmed diagnoses, which may result in an underestimation of the burden of NVAE among medically managed patients and, for this reason, the high mortality rate observed in this review may not accurately represent the true case fatality rate in less severe or non-surgical presentations.

This case and supporting evidence underscore the need to revise existing risk stratification frameworks for fungal endocarditis. From a preventive standpoint, maintaining a high index of suspicion is critical in cirrhotic or immunosuppressed patients presenting with persistent fever and negative blood cultures. Early echocardiographic evaluation and multidisciplinary collaboration may facilitate timely diagnosis. Future efforts should focus on defining intermediate-risk immune phenotypes, validating new diagnostic biomarkers, and developing antifungal strategies that are compatible with multisystem comorbidities. Prospective registries integrating immunological and genomic data are essential for advancing our understanding of this rare but lethal condition. Furthermore, studies in the future using larger datasets or registry-based data could explore temporal and regional trends in more detail.

## 6. Conclusions

Native-valve *Aspergillus* endocarditis remains a diagnostic and therapeutic blind spot in modern infectious diseases. Its rarity belies its lethality, and its evolving epidemiological profile challenges the traditional boundaries of host vulnerability to this pathogen. This study calls for a paradigm shift from rigid definitions of immunosuppression to a more nuanced understanding of immune dysfunction driven by chronic illness, inflammation, or immune remodelling.

In the absence of early clinical suspicion, conventional diagnostic algorithms and therapeutic timing fail. The future of NVAE management will depend not only on technological advances but also on the clinician’s willingness to recognize fungal endocarditis where it was previously unconsidered.

## Figures and Tables

**Table 1:** Summary of the 43 studies included in the systematic review. Each row corresponds to one study (case report or individual case from a case series). Diagnostic confirmation was based on microbiological, histopathological, or molecular testing performed on valvular or embolic tissue. Abbreviations: ABPA: allergic bronchopulmonary aspergillosis; COPD: chronic obstructive pulmonary disease; HIV: human immunodeficiency virus; NR: not reported; PCR: polymerase chain reaction; SFTS: severe fever with thrombocytopenia syndrome; VSD: ventricular septal defect.

Author	Country	Number of Patients	Predisposing Condition	Valve	Diagnosis	Antifungals	Outcome	Surgery
Aggarwal [11]	United States	1	Post-traumatic splenectomy	Mitral	Valvular histology + culture	Amphotericin B	Deceased	Yes
Aldosari [12]	Saudi Arabia	1	Wegener’s granulomatosis	Mitral	Valvular histology + culture	Azole + amphotericin B	Survivor	Yes
Attia [13]	United Kingdom	1	Allogeneic transplant + lymphoma	Aortic	Valvular histology + culture	Azole + amphotericin B	Deceased	Yes
Badiee [14]	Iran	1	None	Aortic	Culture + PCR on valvular tissue	Azole + amphotericin B	Deceased	Yes
Caplan [15]	United States	1	Liver cirrhosis	Aortic	Valvular histology + culture	Amphotericin B	Deceased	No
Chevalier [16]	France	2	Kidney transplant/IgA vasculitis	Aortic/Mitral	Valvular culture only	Not reported/Azole	Deceased	No
Cox [17]	France	1	Kidney transplant	Mitral	Valvular histology + culture	No treatment (postmortem)	Deceased	No
Elzi [18]	Switzerland	1	Kidney transplant	Aortic	Valvular histology + culture	Azole + amphotericin B	Deceased	No
Fayad [19]	France	1	None	Mitral	Valvular culture only	Azole + echinocandin	Survivor	Yes
Fitzpatrick [20]	United States	1	Ulcerative colitis	Mitral	Valvular histology + culture	Azole	Survivor	Yes
Fullin [21]	United States	1	Post-COVID	Mitral	Valvular culture only	Azole	Survivor	Yes
García [22]	Spain	1	HIV	Mitral	Valvular culture only	Not reported	Deceased	No
Gilbey [23]	United States	1	Lung transplant	Mitral	Valvular histology only	Sequential polyene → azole	Deceased	No
Grossman [24]	United States	1	None	Aortic	Valvular culture only	Azole + amphotericin B	Deceased	Yes
Gupta [25]	India	1	COPD	Tricuspid	Valvular histology only	Not reported	Deceased	No
Ikediobi [26]	United States	1	None	Mitral	Valvular histology + culture	Azole + amphotericin B	Deceased	Yes
Jan [27]	United States	1	Leukemia	Aortic	Valvular histology + culture	No treatment (postmortem)	Deceased	Yes
Kanda [28]	Japan	1	Chronic myeloid leukemia	Mitral	PCR on valvular tissue (no culture)	Amphotericin B	Deceased	No
Katsoulis [29]	Australia	1	None	Mitral	Valvular histology + culture	No treatment (postmortem)	Deceased	No
Kuijer [30]	Netherlands	1	Hairy cell leukemia	Aortic	Valvular culture only	Amphotericin B	Deceased	No
Kuroki [31]	Japan	1	Colon cancer	Mitral	Valvular histology + culture	Azole	Survivor	Yes
Maher [32]	United Kingdom	1	Lung transplant	Aortic/Mitral	Valvular culture only	Azole + amphotericin B	Deceased	Yes
Manja [33]	Slovenia	1	Autoimmune hepatitis	Mitral	Valvular histology + culture	Azole	Survivor	Yes
Marín [34]	Spain	1	Kidney transplant	Mitral	Valvular histology + culture	Not reported	Deceased	No
Minhas [35]	India	1	Congenital heart disease (restrictive VSD)	Tricuspid	Valvular histology + culture	Amphotericin B + echinocandin	Survivor	Yes
Najafi [36]	Iran	1	Post-COVID-19	Mitral	Culture + PCR on valvular tissue	Azole + amphotericin B	Deceased	Yes
Ngampongpan [37]	United Kingdom	1	No clinical data available	Aortic	Valvular histology + culture	Amphotericin B	Deceased	Yes
Nusbaum [38]	United States	1	HIV	Mitral	Valvular culture only	Amphotericin B	Survivor	Yes
Palomares [39]	Spain	1	Chronic lymphocytic leukemia	Mitral	Histology + PCR on valvular tissue	Azole	Deceased	No
Pemán [40]	Spain	1	COPD	Mitral	Valvular histology + culture	Azole + echinocandin	Deceased	Yes
Rahman [41]	Pakistan	1	ABPA	Aortic	Valvular histology + culture	Amphotericin B	Survivor	Yes
Regueiro [42]	Spain	1	Lung transplant	Aortic	Valvular culture only	Amphotericin B + echinocandin	Deceased	Yes
Rofaiel [43]	United States	1	Acute promyelocytic leukemia	Mitral	Valvular histology + culture	Amphotericin B monotherapy	Deceased	Yes
Saxena [44]	Australia	1	None	Mitral	Valvular histology + culture	Amphotericin B + echinocandin	Survivor	Yes
Scherer [45]	Germany	1	Lung transplant	Mitral	Valvular histology + culture	Azole + amphotericin B	Deceased	Yes
Sloane [46]	United States	1	Ulcerative colitis	Mitral	Valvular histology + culture	Azole monotherapy	Survivor	Yes
Van Meensel [47]	Belgium	1	Kidney transplant	Tricuspid	Valvular histology + culture	Azole + amphotericin B	Deceased	Yes
Vassiloyanakopoulos [48]	Greece	1	Asthma	Tricuspid	Valvular histology + culture	Azole + amphotericin B	Survivor	Yes
Vohra [49]	United States	1	None	Tricuspid	Valvular histology + culture	Sequential azole → polyene	Deceased	No
Yassin [5]	Iran	1	Rheumatoid arthritis	Mitral	Culture + PCR on valvular/tissue	Azole monotherapy	Survivor	Yes
Zang [50]	China	1	None	Mitral	Combined histology, culture, and PCR on valvular tissue	Azole + echinocandin	Survivor	Yes
Zhao [51]	China	1	SFTS	Aortic	Valvular histology + culture	Azole monotherapy	Survivor	Yes
Zhu [52]	Singapore	1	Type 2 diabetes	Mitral	Valvular histology + culture	Azole + echinocandin	Deceased	Yes

## Data Availability

The original contributions presented in this study are included in the article and Supplementary Material. Further inquiries can be directed to the corresponding author.

## Supplementary Materials

The following supporting information can be downloaded at: https://www.mdpi.com/article/10.3390/jof11120836/s1, PRISMA checklist and PRISMA abstract checklist.

## Abbreviations

The following abbreviations are used in this manuscript:

AE	*Aspergillus* endocarditis
BDG	1,3-β-D-glucan
CAR-T	Chimeric antigen receptor T-cell therapy
CT	Computed tomography
ESBL	Extended-spectrum beta-lactamase
GM	Galactomannan
MRI	Magnetic resonance imaging
NVAE	Native-valve *Aspergillus* endocarditis
PCR	Polymerase chain reaction
TIPS	Transjugular intrahepatic portosystemic shunt
TTE	Transthoracic echocardiography

## Appendix A. Full Search Strategy

Databases searched: PubMed/MEDLINE and Embase

Date of search: 4 March 2025

Search performed using free-text and controlled vocabulary terms.

**PubMed/MEDLINE search strategy:**

*((“Endocarditis”[Mesh]) OR (endocarditi*[Title/Abstract]))*

*AND*

*(((“Aspergillus fumigatus”[Mesh]) OR (aspergillus fumigatus[Title/Abstract]))*

*OR (aspergillus spp[Title/Abstract])*

*OR (a fumigatus[Title/Abstract]))*

**Embase search strategy:** *(‘aspergillus fumigatus’/de OR ‘aspergillus fumigatus’:ab,ti*

*OR ‘aspergillus spp’:ab,ti*

*OR ‘a fumigatus’:ab,ti)*

*AND*

*(‘endocarditis’/exp OR endocarditi*:ab,ti)*

*AND [embase]/lim*

**Search notes:**

- No date or language restrictions were applied.
- References were imported into Rayyan, where duplicate records were automatically identified and removed.
- Two reviewers independently screened titles and abstracts using Rayyan.
- Full-text selection and reference management were performed using Zotero.
- The search strategy was peer-reviewed and designed to ensure reproducibility and sensitivity in identifying relevant literature on Aspergillus native-valve endocarditis.
